# A predictive model for the recurrence of intracranial aneurysms following coil embolization

**DOI:** 10.3389/fneur.2023.1248603

**Published:** 2023-11-14

**Authors:** Tao He, Kun Chen, Ru-Dong Chen

**Affiliations:** ^1^Department of Cardiology, Zhongnan Hospital of Wuhan University, Wuhan, China; ^2^Institute of Myocardial Injury and Repair, Wuhan University, Wuhan, China; ^3^Department of Neurosurgery, Tongji Hospital, Tongji Medical College, Huazhong University of Science and Technology, Wuhan, Hubei, China

**Keywords:** intracranial aneurysm, coil embolization, recurrence, predictive model, diameter maximum, Raymond-Roy occlusion classification, rupture

## Abstract

**Objective:**

This study aimed to identify risk factors for intracranial aneurysms (IAs) recurrence and establish a predictive model to aid evaluation.

**Methods:**

A total of 302 patients with 312 IAs undergoing coil embolization between September 2017 and October 2022 were divided into two groups based on digital subtraction angiography follow-up. Clinical characteristics, operation-related factors, and morphologies were measured. Cox proportional hazard regression was used to identify the risk factors. Hazard ratios (HRs) were used to score points, and a predictive model was established. The test cohorts consisted of 51 IAs. Receiver operating characteristic curves were generated to determine the cutoff values and area under the curves (AUCs). A Delong test was performed to compare the AUCs.

**Results:**

Diameter maximum (D max) (*p* < 0.001, HR = 1.221), Raymond-Roy occlusion classification (RROC) II or III (*p* = 0.004, HR = 2.852), and ruptured status (*p* < 0.001, HR = 7.782) were independent risk factors for the recurrence of IAs. A predictive model was established: D max + 2 ^*^ RROC (II or III; yes = 1, no = 0) + 6 ^*^ ruptured status (yes = 1; no = 0). The AUC of the predictive model (0.818) was significantly higher than those of D max (0.704), RROC (II or III) (0.645), and rupture status (0.683), respectively (Delong test, *p* < 0.05). The cutoff values of the predictive model and D max were 9.75 points and 6.65 mm, respectively.

**Conclusion:**

The D max, RROC (II or III), and ruptured status could independently predict the recurrence of IAs after coil embolization. Our model could aid in practical evaluations.

## Introduction

Intracranial aneurysms (IAs) are a type of cerebrovascular disease that can have a severe impact on an individual's health (1). Coiling embolization is a commonly used and effective method for treating saccular IAs but has been associated with a higher rate of recurrence compared to clipping (2, 3). The recurrence of IAs has become a crucial problem in clinical practice, as it may lead to rebleeding events, treatment-related complications (such as thromboembolic events and intraoperative rupture), and financial burdens (4, 5). Therefore, it is critical to identify aneurysms with high recurrence rates and adjust the related follow-up interval accordingly. Several risk factors associated with recurrence have been demonstrated by some studies, including IAs occurring in the middle cerebral artery (MCA) and posterior circulation, size > 7 mm, stent types, smoking, and ruptured status (2, 6). However, previous studies have only tested the accuracy of independent risk factors with limited model cohorts (2, 6, 7). Thus, we aimed to establish a predictive model to comprehensively explore the risks of IAs recurrence.

## Methods

### Patient and data

This study was approved by our hospital's institutional ethics committee, and we obtained consent from patients or their close relatives before collecting data. From September 2017 to October 2022, a total of 302 patients with 312 aneurysms underwent digital subtraction angiography (DSA) follow-up after coil embolization and were included in this study. Inclusion criteria were as follows: (a) saccular aneurysm; (b) DSA follow-up imaging. Exclusion criteria were as follows: (a) magnetic resonance angiography or computed tomography angiography follow-up imaging; (b) coil or clip treatment history; (c) traumatic, dissecting, or fusiform, blood blister-like aneurysms; (d) refusal to the DSA follow-up.

**Figure 1:**
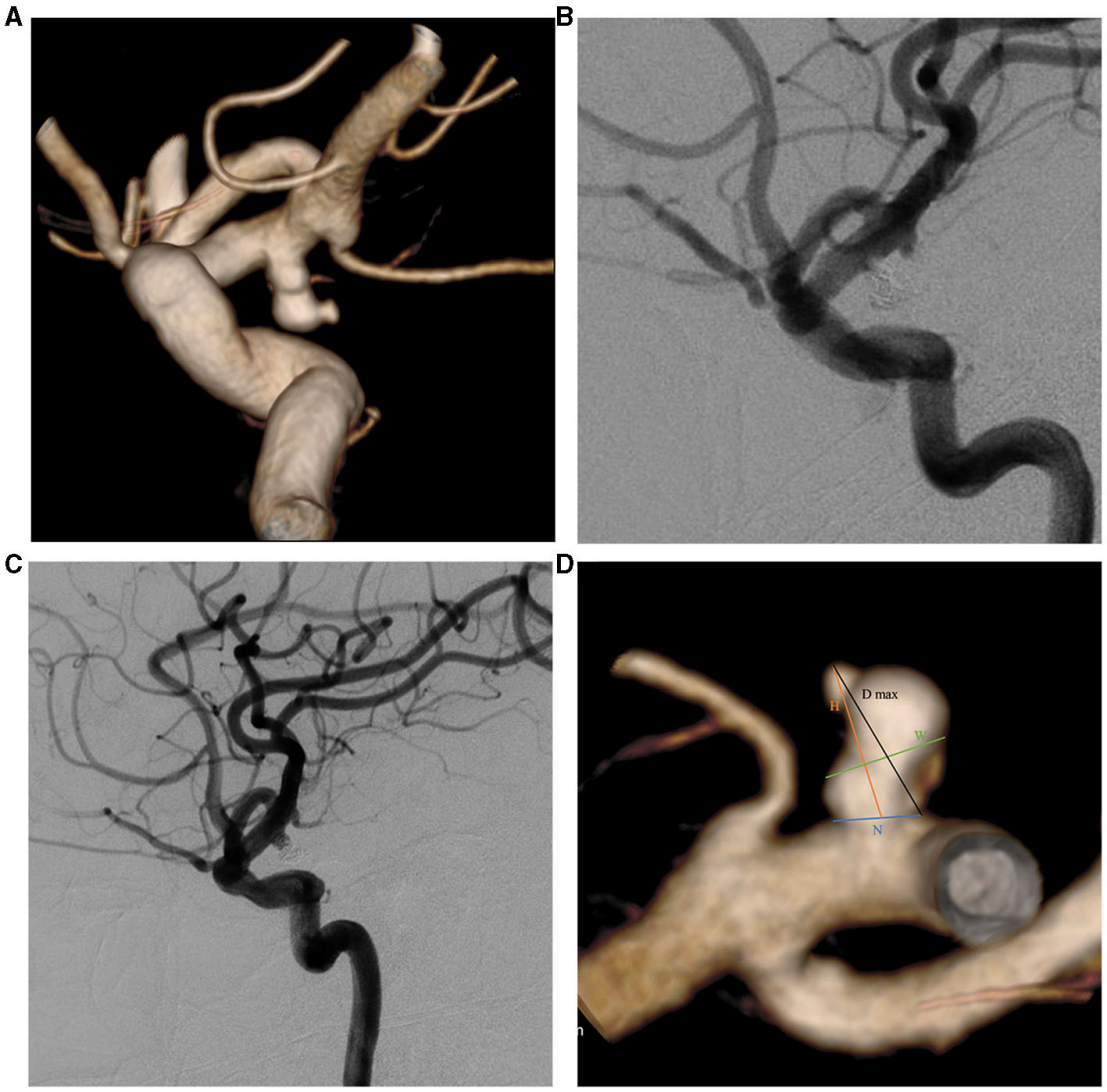
Two-dimensional (2D) and three-dimensional (3D) DSA showing a 49-year-old man with a left PcomA aneurysm. **(A)** 3D-DSA showing a PcomA aneurysm with a daughter sac; **(B)** 2D-DSA showing that the PcomA aneurysm was occluded completely; **(C)** 2D-DSA showing the recurrence signs after 17 months of follow-up; **(D)** morphological parameter definition. D max, diameter maximum; DSA, digital subtraction angiography; H, height; N, neck diameter; PcomA, posterior communicating artery; W, width.

A total of 312 saccular aneurysms were divided into a recurrence group (*n* = 33) and a cured group (*n* = 279). Recurrence was defined as any increase in contrast filling of the aneurysms during follow-up compared with the immediate angiographic outcome after endovascular procedures (Figure 1C) (2, 8). Retreatment criteria included residual aneurysms > 20%, irregular shapes, unstable neck remnants, and high aneurysmal regrowth risks. The angiographic results were evaluated by two experienced neuro-interventional surgeons. Among the 33 recurrent saccular aneurysms, 28 patients underwent coiling retreatment, three underwent clipping, and two decided to follow up. From November 2022 to March 2023, 51 IAs after coil embolization underwent DSA follow-up and were used as test cohorts. Five patients were considered to have recurrent aneurysms without symptoms, and two patients with post-treated IAs experienced re-rupture with subarachnoid hemorrhage.

We collected clinical characteristics, including age, gender, smoking, alcohol consumption, hypertension, diabetes mellitus (DM), hyperlipemia, coronary heart disease (CHD), and cholesterol, triglyceride, and glucose levels. We also recorded operation-related data, such as ruptured status, aneurysm location, high seniority, operation time, stent types (coil alone, braided, and laser-cut type), Raymond-Roy occlusion classification (RROC) (grade I and grade II or III), and aneurysm morphologies. Aneurysm location was divided into five parts, namely, internal carotid artery, posterior communicating artery (PcomA), MCA, anterior cerebral artery, and posterior circulation (vertebral, posterior inferior cerebellar artery, superior cerebellar artery, and basilar artery). High seniority was defined as surgeons with more than 10 years of experience. Roy et al.'s study (8) was used to describe RROC. RROC grade I represented complete embolization without any contrast filling of the aneurysm; grade II was defined by a residual neck (<2 mm), and grade III was defined by a residual part of the aneurysm sac. Stent types were categorized based on stent usages, and all patients with stents took routine dual antiplatelet therapy (combination of Aspirin 100 mg/day with clopidogrel 75 mg/day or ticagrelor 90 mg bid).

### Morphological parameter measurement

Pretreatment DSA images and three-dimensional reconstructions were obtained for each patient using the Innova Workplace system (GE Medical) (Figures 1A, B). Two experienced neuro-interventional surgeons performed measurements of morphological parameters using the same procedure, and we defined the parameters as the average of their values. The results were precise to two decimal places. The morphological variables were defined as follows (Figure 1D):

(1) Maximum diameter (D max): The maximum diameter of the aneurysm;

(2) Height (H): The maximum distance from the center of the aneurysm neck to a point on the sac;

(3) Width (W): The maximum distance perpendicular to H.

(4) Neck diameter (N): The maximum diameter in the neck plane.

(5) Parent artery diameter (PD): The average value of vessel diameters at the proximal and distal sites.

(6) Bottleneck factor (BNF): The ratio of W to N.

(7) H/W: The ratio of H to W.

(8) Size ratio (SR): The ratio of H to the PD.

(9) Aspect ratio (AR): The ratio of H to N.

(10) Daughter sac: whether or not there is a daughter sac connected to the aneurysmal sac.

### Statistical analysis

Statistical analysis was performed using SPSS 20.0 (IBM Inc., Chicago, IL). The normality of the data was determined using the Shapiro-Wilk test. Continuous variables with a normal distribution were expressed as mean ± standard deviation, while those without were expressed as median ± interquartile range. Categorical variables were reported as frequencies (percentages). A non-parametric test was used to analyze the follow-up time between the two groups. We performed Cox proportional hazard regression to test all variables in the univariate analysis. Using the stepwise forward method, we conducted multivariate Cox regression analysis, which involved the following significant parameters from the univariate analysis: triglyceride, glucose, stent types, location, ruptured status, RROC, H, W, D max, N, PD, and SR. Based on the independent risk factors, we established a predictive model and assigned predictive scores using hazard ratios (HRs). Receiver operating characteristic (ROC) curves corresponding to the independent risk factors and the predictive model were generated to derive their respective areas under the curves (AUCs) and cutoff values. The Delong test was performed to compare the AUCs. Differences where *p* < 0.05 were statistically significant.

## Results

### Intracranial aneurysm recurrence rates

Of the 302 patients included in the study, 10 had two aneurysms that were successfully treated with endovascular therapy. The mean age of the patients was 53.66 years, and there were 221 female patients. In total, 312 aneurysms were analyzed, including 33 that had recanalized and 279 that had not. Of these aneurysms, 213 (68%) were classified as RROC I, and 99 (32%) were classified as RROC II or III. The overall recurrence rate was 11%, and the average follow-up period was 12.53 months. There was no significant difference in follow-up time between the two groups (*p* = 0.124).

Among the 106 ruptured aneurysms, the recurrence rate was 21%, with 31 (29%) cases classified as RROC II or III. Among the 78 aneurysms treated with coiling alone, the recurrence rate was 23%. Of the 106 aneurysms treated with braided stents, the recurrence rate was 8%, with 99 (93% of cases) using Lvis and 7 (7% of cases) using Leo. Of the 128 aneurysms treated with laser-cut stents, the recurrence rate was 5%, with 123 (96%) cases using Enterprise, 4 (3%) cases using Atlas, and 1 (1%) case using Solitare. In the univariate Cox regression analysis, braided (*p* = 0.002, HR = 0.267) and laser-cut (*p* = 0.001, HR = 0.238) stents were significantly associated with a lower risk of recurrence. However, the difference in recurrence rates between braided and laser-cut stents was not statistically significant (*p* = 0.825).

### Risk factors for the IAs recurrence

The univariate analysis of clinical characteristics, aneurysm morphologies, and operation-related variables is presented in Table 1. In the univariate Cox regression analysis, the recurrence group had significantly higher levels of triglycerides (1.11 vs. 1.10 mmol/L, *p* = 0.003) and glucose (5.57 vs. 5.17 mmol/L, *p* = 0.047) compared to the cured group. Gender, age, hypertension, hyperlipemia, DM, CHD, smoking, alcohol, high seniority, and cholesterol showed insignificant differences (*p* > 0.05). Among the operation-related variables, the recurrence group had a higher percentage of aneurysms at ruptured status (67% vs. 30%, *p* < 0.001) and RROC II or III (58% vs. 29%, *p* = 0.001), located at PcomA (36% vs. 14%, *p* = 0.002), and posterior circulation (42% vs. 26%, *p* = 0.015). It is worth noting that recurrent aneurysms had a lower percentage of stent usage, and stents appeared to play a protective role. However, it is important to highlight that there was no statistically significant difference between braided stents and laser-cut stents (*p* > 0.05). However, the operation time was not associated with aneurysm recurrence (*p* = 0.404). In the aneurysm morphologies, the recurrence group had significantly smaller PD (3.12 vs. 3.50 mm, *p* = 0.028) and larger H (6.79 vs. 4.10 mm, *p* = 0.001), W (5.40 vs. 4.40 mm, *p* = 0.008), D max (8.77 vs. 5.50 mm, *p* < 0.001), N (4.80 vs. 4.10 mm, *p* = 0.005), and SR (2.23 vs. 1.26, *p* < 0.001) compared to the cured group. On the other hand, daughter sac, H/W, BNF, and AR show insignificant associations with aneurysm recurrence (*p* > 0.05). Besides, we further identify the significant predictors for recurrence in ruptured and unruptured aneurysms as mentioned in Table 2.

**Table 1:** Univariate analysis of clinical characteristics, aneurysm morphology, and operation-related factors.

**Variables**	**Recurrence group (*n* = 33)**	**Cured group (*n* = 279)**	***p*-value**
Female	24 (73%)	204 (73%)	0.853
Age	54.00 ± 12.00	54.00 ± 11.00	0.277
Hypertension	14 (42%)	126 (45%)	0.889
Hyperlipemia	4 (12%)	17 (6%)	0.253
Diabetes mellitus	0 (0%)	13 (5%)	0.429
CHD	3 (9%)	24 (9%)	0.649
Smoking	4 (12%)	30 (11%)	0.665
Alcohol	3 (9%)	31 (11%)	0.784
Cholesterol, mmol/L	4.15 ± 1.28	4.24 ± 1.23	0.308
Triglyceride, mmol/L	1.11 ± 0.44	1.10 ± 0.46	**0.003**
Glucose, mmol/L	5.57 ± 1.30	5.17 ± 1.07	**0.047**
Follow-up time, months	12.00 ± 6.00	12.00 ± 6.00	0.124
Location			**0.033**
ICA	6 (18%)	135 (48%)	Reference
PcomA	12 (36%)	40 (14%)	**0.002**
MCA	1 (3%)	22 (8%)	0.778
ACA	0 (0%)	10 (4%)	0.980
Posterior circulation	14 (42%)	72 (26%)	**0.015**
High seniority	24 (75%)	156 (55.91%)	0.209
Operation time, min	95.00 ± 43.00	89.00 ± 44.00	0.404
Stent types			0.001
Without stents	18 (55%)	60 (22%)	Reference
Braided	8 (24%)	98 (35%)	**0.002**
Laser-cut	7 (21%)	121 (43%)	**0.001**
Ruptured status	22 (67%)	84 (30%)	**< 0.001**
RROC II or III	19 (58%)	80 (29%)	**0.001**
**Morphology**			
Daughter sac	14 (42%)	98 (35%)	0.448
Height, mm	6.79 ± 3.41	4.10 ± 2.40	**0.001**
Width, mm	5.40 ± 4.90	4.40 ± 2.30	**0.008**
Height/Width	1.00 ± 0.54	0.94 ± 0.33	0.113
D max, mm	8.77 ± 4.06	5.50 ± 3.00	**< 0.001**
Neck diameter, mm	4.80 ± 4.50	4.10 ± 2.20	**0.005**
Bottleneck factor	1.08 ± 0.20	1.03 ± 0.32	0.685
PD, mm	3.12 ± 0.80	3.50 ± 1.30	**0.028**
Size ratio	2.23 ± 1.02	1.26 ± 1.08	**< 0.001**
Aspect ratio	1.15 ± 0.39	0.96 ± 0.57	0.440

p < 0.05 are mentioned in bold.

ACA, anterior cerebral artery; CHD, coronary heart disease; D max, diameter maximum; ICA, internal carotid artery; MCA, middle cerebral artery; PcomA, posterior communicating artery; PD, parent artery diameter; RROC, Raymond-Roy occlusion classification.

**Table 2:** Significant level of risk factors for recurrence in the ruptured and unruptured aneurysms.

**Variables**	**Ruptured aneurysms (*n* = 106)**	**Unruptured aneurysms (*n* = 206)**
Female	0.277	0.659
Age	0.235	0.799
Hypertension	0.200	0.249
Hyperlipemia	0.957	0.147
Diabetes mellitus	0.604	0.647
CHD	0.500	0.789
Smoking	0.682	0.483
Alcohol	0.421	0.750
Cholesterol, mmol/L	0.758	0.507
Triglyceride, mmol/L	0.052	0.476
Glucose, mmol/L	0.884	0.881
Location	0.722	0.558
ICA	Reference	Reference
PcomA	0.342	0.166
MCA	0.997	0.991
ACA	0.984	0.994
Posterior circulation	0.998	0.102
High seniority	0.272	0.903
Operation time, min	0.576	0.171
Stent types	0.888	**0.002**
Without stents	Reference	Reference
Braided	0.658	**0.002**
Laser-cut	0.761	**0.004**
RROC II or III	**0.012**	**0.047**
**Morphology**		
Daughter sac	0.430	0.225
Height, mm	**0.009**	**0.005**
Width, mm	**< 0.001**	0.147
Height/Width	**0.038**	**< 0.001**
D max, mm	**< 0.001**	**0.014**
Neck diameter, mm	**< 0.001**	0.114
Bottleneck factor	0.310	0.960
PD, mm	0.937	0.064
Size ratio	0.235	**0.002**
Aspect ratio	**0.043**	**0.006**

p < 0.05 are mentioned in bold.

ACA, anterior cerebral artery; CHD, coronary heart disease; D max, diameter maximum; ICA, internal carotid artery; MCA, middle cerebral artery; PcomA, posterior communicating artery; PD, parent artery diameter; RROC, Raymond-Roy occlusion classification.

**Figure 2:**
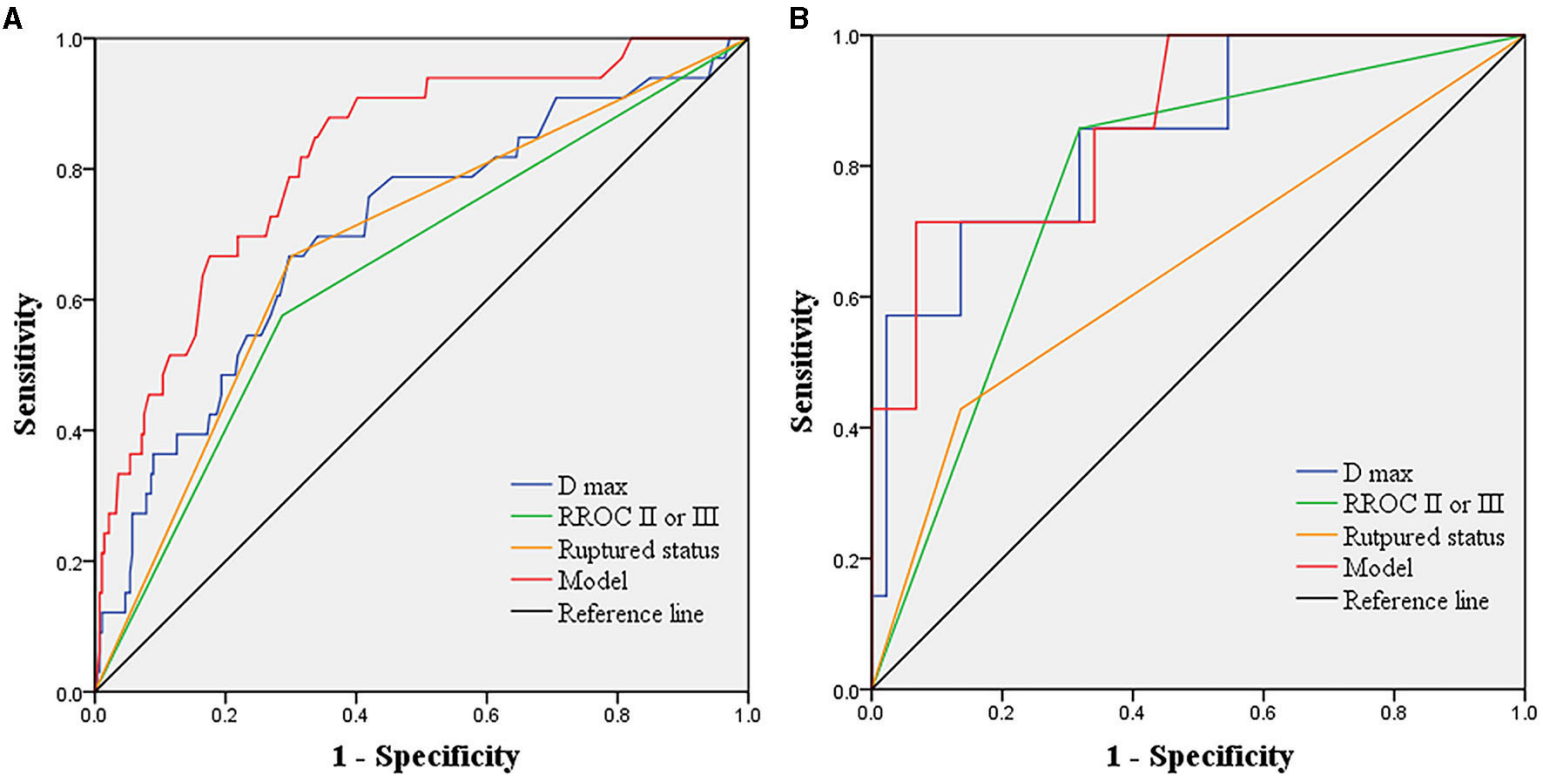
Receiver operating characteristics analysis showing the predictive performance of the model and independent risk factors in the derived cohorts and test cohorts. **(A)** in the derived cohorts, AUCs of the predictive model, D max, RROC II or III, and ruptured status are 0.818, 0.704, 0.645, and 0.683, respectively. **(B)** in the test cohorts, AUCs of the predictive model, D max, RROC II or III, and ruptured status are 0.869, 0.847, 0.769, and 0.646, respectively. AUCs, area under the curves; D max, diameter maximum; RROC, Raymond-Roy occlusion classification.

We conducted multivariate Cox regression analysis using the stepwise forward method to identify independent predictors of recurrence (Table 3). D max (*p* < 0.001, HR = 1.221, CI = 1.111–1.342), RROC II or III (*p* = 0.004, HR = 2.852, CI = 1.385–5.871), and ruptured status (*p* < 0.001, HR = 7.782, CI = 3.428–17.667) were determined as independent risk factors. A predictive model was established based on the independent risk factors. Hazard ratios (HRs) were used to allocate the predictive scores (Table 3). The ratio of HRs of RROC and rupture status to the HR of D max was 2.34 and 6.37, respectively. Since the ratio was not convenient to calculate, we assigned D max, RROC II or III, and rupture status as 1, 2, and 6 points, respectively. Therefore, the predictive model was as follows: 1^*^D max + 2^*^ RROC II or III (yes = 1; no = 0) + 6^*^ rupture status (yes = 1; no = 0). The results of the ROC analysis and Delong test are shown in Table 4. The AUCs of the predictive model performed better than D max, RROC II or III, and rupture status (Delong test, *p* < 0.05, 0.818 vs. 0.704, 0.645, and 0.683, respectively) (Figure 2A). Moreover, the cutoff values of the prediction model and D max were 9.75 points and 6.65 mm, respectively. Finally, the test cohort consisted of 51 patients to verify the performance of our predictive model. The AUCs of the predictive model, D max, RROC II or III, and rupture status were 0.869, 0.847, 0.769, and 0.646, respectively (Figure 2B).

**Table 3:** The results of multivariate Cox regression analysis and predictive scores.

**Variables**	**β**	***p***	**HR**	**95% CI**	**Scores**
D max	0.200	< 0.001	1.221	1.111–1.342	1
RROC II or III	1.048	0.004	2.852	1.385–5.871	2
Ruptured status	2.052	< 0.001	7.782	3.428–17.667	6

D max, diameter maximum; CI, confidence interval; HR, hazard ratio; RROC, Raymond-Roy occlusion classification.

**Table 4:** The results of ROC analysis and Delong test.

**Variables**	**AUC**	**SE**	**95% CI**	***P*-value (Delong test)**
Predictive model	0.818	0.038	0.743–0.893	
Ruptured status	0.683	0.050	0.585–0.781	0.002
RROC II or III	0.645	0.053	0.541–0.748	0.002
D max	0.704	0.051	0.605–0.804	0.003

The Delong test was performed to compare the predictive model with other variables.

AUC, area under the curves; CI, confidence interval; D max, diameter maximum; ROC, receiver operating characteristics; RROC, Raymond-Roy occlusion classification; SE, standard error.

## Discussion

The coil embolization has been determined to be an effective method to treat IAs ^5^. However, recurrence is a clinical challenge that can lead to re-hemorrhage, more challenging treatment, operation-related risks, and a heavy financial burden for patients. The Barrow Ruptured Aneurysm Trial and International Subarachnoid Aneurysm Trial have reported the retreatment rate after coil embolization to be up to 19% and 17%, respectively (9, 10), which is higher than after microsurgical clipping (11). Despite continued improvement in related techniques and devices, the potential for recurrence still remains (7). Risk factors linked to recurrence have been widely studied through comparative analysis, showing the significance of smoking, location, subarachnoid hemorrhage, aneurysm size, and neck with the recurrence rate (2, 7, 12). However, some studies neglect the influence of follow-up time in the analysis (12, 13). Moreover, there have been few models to comprehensively evaluate aneurysms at high risk of recurrence. Therefore, we analyzed the clinical characteristics, operation-related factors, aneurysm morphologies, and laboratory parameters of patients with IAs using the Cox proportional hazard regression analysis. The recurrence rate was 11%, which is close to the 10% reported in a multicenter study (2). Furthermore, our predictive model outperformed any independent predictors. The AUCs of the predictive model, D max, RROC II or III, and ruptured status were 0.818, 0.704, 0.645, and 0.683, respectively.

Our study's findings are consistent with previous studies, indicating that a higher D max is associated with a greater risk of recurrence in most centers (2, 14). Larger aneurysms require more coiling to prevent excess blood flow into the aneurysm, which can affect thrombosis and recurrence rates (2). In our study, the mean and cutoff values of D max for recurrent aneurysms were 8.77 mm and 6.65 mm, respectively. Additionally, follow-up time was related to recurrence rates. Early recanalization was positively correlated with increasing D max, particularly for values >7 mm, while complete aneurysm occlusion rates were negatively correlated with D max values < 7 mm (7). D max has been demonstrated to have predictive value for ruptured aneurysms, and the rupture status can independently predict aneurysm recurrence. Based on clinical experience, neurosurgeons tend to regard IAs with a larger N as being associated with a high risk of recurrence (15). However, there have been limited studies regarding the significance of N in multivariate analysis. Our previous study showed that larger N provided a protective role in preventing aneurysm rupture, but the ruptured status could increase the risk of recurrence. Furthermore, statins can inhibit matrix metalloproteinases to lower the recurrence rate in aneurysms with D max <10 mm (16). In the Murayama et al. study, aneurysms with a D max >10 mm had a high recurrence rate of 35% (17). In Chalouhi et al.'s study of stent-assisted coiling, larger aneurysms, previously coiled aneurysms, the use of neuroform stents, and aneurysm location were emphasized as having predictive value for recurrence, which is consistent with our results in univariate analysis (18).

After coil embolization, ruptured aneurysms are more likely to develop recurrence due to the disturbed coil distribution caused by the condition of the aneurysm sac. The surgeon might insert fewer coils to avoid intraoperative hemorrhage caused by coil perforation. For instance, the surgeons may protect the PcomA by sparsely coiling the neck in a PcomA aneurysm, which aligns with the aneurysm location identified in univariate analysis. In our study, 29% of the 106 ruptured aneurysms had RROC II or III, which is indicative of the recurrence of the aneurysm. Stent-assisted treatment is controversial for ruptured aneurysms due to concerns regarding safety and efficacy. Stents can alter hemodynamics, induce new intimal hyperplasia, and result in remodeling of the parent artery. Our results are consistent with other studies that suggest braided and laser-cut stents are negatively correlated with recurrence. Dynamic instability and clot lysis in the aneurysmal sac may also contribute to recurrence (7). Besides, stent usage was significant in unruptured aneurysms but insignificant in ruptured aneurysms. Changes in aneurysm morphology after rupture can lead to changes in volume and thrombolysis, further increasing the risk of aneurysm recurrence. Yi et al. stressed that Neuroform Atlas stents have been shown to have a higher incidence of complete occlusion and lower rates of recurrence in ruptured IAs (19). RROC II (remnant neck) was not uncommon in clinical practice, with reported rates of 20%−60% (20). Among 312 aneurysms, 99 (32%) cases were at RROC II or III (86 cases were grade II, and 13 were grade III) in our study. Stephan et al.'s study followed up on 625 RROC II or III IAs treated by coiling or stent-assisted coiling and demonstrated that older age, aneurysm size, and ruptured status have been associated with rupture, but unruptured aneurysms at RROC II only have a 1% rupture rate (20). RROC has been determined to be a valuable variable in predicting the recurrence of IAs, as highlighted by various studies. For instance, Darflinger et al. reported a significant correlation between the initial RROC and retreatment in a sample of 4,587 IAs (21). However, Mascitelli et al.'s study showed that there was no statistically significant difference in recurrence rates between RROC I and II (22). These findings suggest that a comprehensive evaluation of independent risk factors may be necessary for more accurate predictions.

Current models for predicting aneurysm recurrence are limited. Lin et al. explored the short-term recurrence model in 6 months based on significant variables in the univariate analysis (15). We developed a novel predictive model based on independent risk factors, with an average follow-up of 12.53 months. Our combination model integrating D max, RROC, and ruptured status outperformed any single variable, with an AUC of 0.818 compared to 0.704, 0.645, and 0.683, respectively. External tests confirmed the model's robustness. While the HR values generated accurate predictions, their clinical utility was limited by practical considerations. To simplify this process, we scored D max, RROC II or III, and ruptured status as 1, 2, and 6 points, respectively. By summing these scores, we identified IAs with the highest risk of recurrence. When the total scores exceeded the cutoff value of the prediction model (9.75 points), a short-term DSA follow-up was recommended for patients. Our model represents a significant step forward in predicting aneurysm recurrence.

This study has several limitations that should be taken into consideration. First, its retrospective design introduces a risk of bias and confounding factors. Second, the sample sizes of the derived and test cohorts were limited due to the study being conducted at a single institute. Follow-up was done through telephone consultations, non-invasive angiography in outpatient settings, and DSA in hospitals, with the patients' consent. Third, the modified ROCC was not applied as there were only 13 patients at Grade III, and further classification was not necessary. Fourth, the limited number of stent types used prevented more comprehensive categorization. Fifth, we did not involve the flow diverter due to a lack of angiographic follow-up and limited patient numbers. Sixth, we did not include hemodynamic parameters in our analysis, and computer fluid dynamics could provide a more accurate analysis (23). However, it is important to note that some studies have indicated that morphology can affect the distribution and magnitude of WSS, leading to biased effect estimations (24, 25). Therefore, future multicenter prospective studies are necessary to evaluate the predictive performance of our model in the recurrence of intracranial aneurysms.

## Conclusion

Our study revealed that D max, RROC II or III, and ruptured status are independent risk factors for the recurrence of intracranial aneurysms after coil embolization. By integrating these independent risk factors, our predictive model can provide comprehensive assistance in practical evaluations. This research adds to the growing body of evidence on risk factors for IA recurrence and provides a useful tool to identify high-risk patients and provide timely intervention.

## Data availability statement

The raw data supporting the conclusions of this article will be made available by the authors, without undue reservation.

## Ethics statement

This study was reviewed and approved by institutional Ethics Committee at Tongji Hospital. Written informed consent was obtained from all participants or their legal guardians/next of kin for their participation in this study.

## Author contributions

TH wrote the draft. KC collected and analyzed data. R-DC designed, revised, and supervised the study. All authors contributed to the article and approved the submitted version.
